# Integration of DNA Repair, Antigenic Variation, Cytoadhesion, and Chance in *Babesia* Survival: A Perspective

**DOI:** 10.3389/fcimb.2022.869696

**Published:** 2022-04-14

**Authors:** David R. Allred

**Affiliations:** ^1^Department of Infectious Diseases and Immunology, University of Florida, Gainesville, FL, United States; ^2^Genetics Institute, University of Florida, Gainesville, FL, United States; ^3^Emerging Pathogens Institute, University of Florida, Gainesville, FL, United States

**Keywords:** antigenic variation, *Babesia*, cytoadhesion, DNA Repair, immune evasion, oxidative stress

## Abstract

Apicomplexan parasites live in hostile environments in which they are challenged chemically and their hosts attempt in many ways to kill them. In response, the parasites have evolved multiple mechanisms that take advantage of these challenges to enhance their survival. Perhaps the most impressive example is the evolutionary co-option of DNA repair mechanisms by the parasites as a means to rapidly manipulate the structure, antigenicity, and expression of the products of specific multigene families. The purpose of variant proteins that mediate cytoadhesion has long been thought to be primarily the avoidance of splenic clearance. Based upon known biology, I present an alternative perspective in which it is survival of the oxidative environment within which *Babesia* spp. parasites live that has driven integration of DNA repair, antigenic variation, and cytoadhesion, and speculate on how genome organization affects that integration. This perspective has ramifications for the development of parasite control strategies.

## Introduction

Many unsuccessful years of effort aimed at continuous *in vitro* cultivation of *Plasmodium falciparum* came to fruition in 1976 at the Trager Laboratory of Rockefeller University. A key element turned out to be the fortuitous use of a “candle jar” to provide the elevated CO_2_ atmosphere assumed to be needed for growth, which also had lowered the O_2_ tension (Trager and Jensen, 1976). When parasites were found to continue replicating it was realized, and later established, that *P. falciparum* is a microaerophile (Scheibel et al., 1979). This lead was followed in establishing an *in vitro* culture system for the related hemoparasite, *Babesia bovis*, which also thrives under microaerophilic conditions (Levy and Ristic, 1980). This trait may be important in unobvious ways.

The shared trait of microaerophilia dovetails with another bit of shared biology in these two species: infected-erythrocytes (IE) of each species cytoadhere to the capillary and post-capillary venous endothelium of major organs [reviewed elsewhere (Allred and Al-Khedery, 2004)]. Cytoadhesion is thought to mediate avoidance of IE passage through the spleen, where disruption of normal erythrocyte membrane characteristics and rheologic properties might result in their removal (Berendt et al., 1994; Allred et al., 2003). However, it is likely that cytoadhesion also provides the opportunity to complete developmental maturation and genome replication under hypoxic conditions. While all *Babesia* and *Plasmodium* parasites studied to date benefit from reduced oxygen tension (Trager and Jensen, 1976; Levy and Ristic, 1980; Vega et al., 1985; Takagi and Waki, 1987; Long et al., 1989; Zweygarth et al., 1995; Chotivanich et al., 2001; Sunaga et al., 2002; McCormack et al., 2019), only a few are known to cytoadhere or sequester as blood stages. If hypoxic conditions during development are not essential, then why does cytoadhesion occur at all? I propose for consideration that the answer is the intersection of DNA repair, cytoadhesion, antigenic variation … and chance.

## Why Cytoadhesion?

Cytoadhesion in *B. bovis* is mediated through the VESA1 protein (O’Connor and Allred, 2000), a heterodimeric protein with two subunits, VESA1a and 1b (Allred et al., 1993; Allred et al., 1994; O’Connor et al., 1997). By contrast, in *P. falciparum* this function is mediated by a monomeric protein, PfEMP1 (Baruch et al., 1995; Smith et al., 1995; Su et al., 1995). Despite the shared function and similar ecological niches occupied by each species, the proteins mediating this function and the genes encoding them are unrelated. They do, however, share the trait of being members of large multigene families that arose through amplification and diversification of progenitor genes. Among the *Babesia sensu stricto*, recognizable *ves* multigene families encoding VESA1-related proteins are consistently present but have undergone further evolutionary innovation and functional diversification into at least two unique branches in each species (Jackson et al., 2014). In *B. bovis*, this diversity is manifested by the two major branches, *ves1*α and *ves1*β, together comprised of over 110 members, and a third branch of three *ves1*γ genes. Other *Babesia* spp. are not known to undergo ligand-mediated cytoadhesion. Why are these amplified gene families retained when most species do not cytoadhere? The answer may be that cytoadhesion has repeatedly arisen independently during speciation in different parasite lineages. In the case of *Babesia* spp. the *ves* gene families of different species all differ significantly in sequence and sometimes structure, yet are consistent in these traits within a species. Despite these differences there is widespread synteny among *Babesia* spp. in chromosomal regions inhabited by *ves* loci. This indicates the presence of amplified *ves* loci in a common ancestor (Jackson et al., 2014), with subsequent innovation and selection, including evolution of *ves* gene sequences and structures capable of mediating cytoadhesion in *B. bovis* during or after speciation. By definition, speciation is the outcome of having arrived at different evolutionary solutions to survival challenges. One cannot expect all species to cytoadhere if this trait was not present in the progenitor, and most do not. It is significant that cytoadhesion also arose in *P. falciparum* and closely related species, whereas other *Plasmodium* spp. do not share *var* genes or cytoadhere.

How could cytoadhesion provide sufficient selection for it to repeatedly arise independently? The evolution of cytoadhesion in *B. bovis* was accompanied by formation of abnormal ridge-like structures in the IE membrane at which VESA1 cytoadhesion ligands are assembled (Aikawa et al., 1997; O’Connor and Allred, 2000; Xiao et al., 2010). As most VESA1 protein isoforms are not adhesive these ridge-like modifications would increase IE removal by the spleen and would be deleterious if they did not serve some beneficial purpose (O’Connor et al., 1999). Despite ready availability of many nutrients in the plasma, to live within a red blood cell is to survive in a harsh, difficult environment. Intraerythrocytic parasites must contend with significant challenges, such as surviving frequent rheologic contortions, obtaining nutrients despite being surrounded by membranes of host origin, and modifying those membranes to serve the parasite’s purposes. One major challenge is simply living within a pool of hemoglobin (Hb). The parasite first must compete with Hb for O_2_ needed for its own metabolism (Barry, 1984). As plasma pH drops within hypoxic tissues the parasite is then inundated with free O_2_ given up by Hb. As the O_2_ diffuses in all directions the parasite lives in the line of fire. During the brief bouts of high O_2_ partial pressure oxidative damage may occur to many components, perhaps exacerbated by hemoglobin peroxidase activity and associated radicals (Reeder, 2017; Zhang et al., 2020). It is clear that appreciable oxidative damage occurs, based upon the accumulation of the lipid oxidation product, malondialdehyde, in IE membranes and fragmentation of DNA (Commins et al., 1988; Murase et al., 1996; Esmaeilnejad et al., 2020), challenging parasite genome integrity. Malondialdehyde is itself carcinogenic, forming DNA base adducts (Marnett, 2000). Damage generated by reactive oxygen species or nitrosylation from innate immune responses also would contribute to selective pressure (Asada et al., 2015; Zhang et al., 2020). Cytoadhesive parasites avoid both clearance by the spleen and genotoxic damage from O_2_ acquired in the lungs. The advantage of enhanced genome integrity may have offset the accompanying enhanced susceptibility to splenic removal. Therefore, both the spleen and lungs would be applying different selective pressures, in a synergistic manner.

## How Do the Parasites Respond to Genotoxic Stress?

Oxidation damage occurring to DNA typically occurs in the form of oxidized bases, most commonly 8-oxo-7,8-dihydroguanine (8-OG), although many forms are possible (Merta et al., 2019; Kumar et al., 2020). Reactive nitrogen species (RNS) may also damage DNA and affect parasite viability, including through direct deamination (Dong and Dedon, 2006; Ohshima et al., 2006; Asada et al., 2015; Li et al., 2017). Damaged bases must be replaced in order to allow replication to continue and to maintain integrity of the genome (Radak and Boldogh, 2010; Garcia-Rodriguez et al., 2018; Kumar et al., 2020). Mechanisms for base replacement involve a single- and often double-stranded DNA break- a potentially lethal event. Repair of these breaks is essential to maintaining genome integrity. Viewed differently, the cycles of damage and repair provide opportunities for loosely targeted sequence modifications during the repair process, an important point to which I will return.

Beyond sparse experimental evidence and what can be gleaned from genomic sequences, little is known about DNA repair among apicomplexan parasites, other than it is a robust example of the adage, “less is more”. For example, canonical non-homologous end-joining (cNHEJ) is a major repair process in higher eukaryotes (Rulten and Grundy, 2017) and would seem essential for haploid asexual parasites. Yet, while present in *Toxoplasma gondii*, most or all of the enzymatic machinery for cNHEJ is absent in *Babesia* and most apicomplexan parasites (Fox et al., 2011; Mack et al., 2019; Nenarokova et al., 2019). Homologous recombination (HR), another major repair process in eukaryotes, is also not utilized equivalently by *B. bovis*. Rad51 (and related) proteins are integral to HR (Symington et al., 2014), yet knock-out of the *B. bovis* Bb*rad51* gene surprisingly had no effect on *in vitro* parasite survival, growth rate, or chromosome reassembly kinetics following γ-irradiation. Although the sensitivity phenotype of Bb*rad51* knockouts for the alkylating agent, methylmethane sulfonate, is consistent, it is not large (Mack et al., 2019). Loss of BbRad51 does, however, eliminate the ability to recombine exogenous DNA into the *B. bovis* genome, an HR form of recombination (Mack et al., 2019). Thus, the overall *in vitro* viability of parasites is unaffected by simultaneous natural and induced lack of cNHEJ and HR, respectively, suggesting that alternative mechanisms are robust at genome maintenance in the absence of overt insult.

## Cytoadhesion and DNA Repair Are Related?

It was recognized more than 50 years ago that the surface of *B. bovis*-IE is antigenically distinct from that of uninfected erythrocytes (Callow, 1968). This difference is due at least in part to parasite-derived proteins integrated into the IE membrane (Allred et al., 1993). Cytoadhesion depends upon parasite-derived components that are targets of host adaptive immunity. In an *in vitro* assay antibody recognition of the cytoadhesion ligand, the protein VESA1, was found to block or reverse this function (O’Connor and Allred, 2000). Under constant hydrodynamic shear *in vivo* this presumably would result in the IE re-entering the circulation covered in antibody, and removal by the spleen (Allred, 1995). This outcome was demonstrated directly for *P. falciparum in vivo* cytoadhesion and sequestration in *Aotus* monkeys (David et al., 1983). In the case of *B. bovis* the antigenicity of VESA1 was found to vary clonally over brief periods in the infected host, reflecting rapid antigenic variation (Allred et al., 1994). This finding led to identification of the *ves* multigene family encoding VESA1 polypeptides (Allred et al., 2000; Xiao et al., 2010), and determination that variation is achieved through a process of segmental gene conversion (SGC) (Al-Khedery and Allred, 2006). During SGC short segments (109 bp on average) of the actively transcribed *ves* genes are replaced with alternative sequences from silent *ves* genes in loci scattered about the genome (Al-Khedery and Allred, 2006; Mack et al., 2020). The outcome of this back-and-forth between recognition and destruction versus variation and escape is long-term persistence with wide population fluctuations (Allred et al., 1994; Calder et al., 1996). Not surprisingly, cytoadhesive function varies along with ligand antigenicity (O’Connor and Allred, 2000), and selection by survival may determine the major expressed isoform within the parasite population.

The *ves* multigene family is large and scattered over all *B. bovis* chromosomes (Al-Khedery and Allred, 2006; Brayton et al., 2007), yet only one *ves* locus (comprised of one *ves1*α and one *ves1*β) is transcriptionally active at a time, the locus of active *ves* transcription (LAT) (Al-Khedery and Allred, 2006; Żupańska et al., 2009; Mack et al., 2020). Importantly, transcriptionally active chromatin is more susceptible than silent chromatin to adoption of oxidation-prone higher-order structure, damage, and mutation (Makova and Hardison, 2015). It remains unclear how such damage is repaired, but we propose that the observed unidirectional movement of duplicated sequence patches that identifies SGC reflects the repair process. In most instances SGC appears as though it represents canonical HR, but this was not supported by knock-out of the Bb*rad51* gene, which had little effect on SGC (Mack et al., 2019; Mack et al., 2020). As the Rad51 superfamily is pivotal to HR throughout all three biological Kingdoms (Jiang et al., 2018) this outcome raises real doubt that SGC is a product of classical HR. While the underlying mechanisms are unclear, the result of SGC is a seemingly endless variety of slightly modified alternative versions of the actively transcribed *ves* genes and the proteins they encode.

As a mechanism to create structural and antigenic diversity of VESA1 proteins, SGC seems ideal. VESA1 has minimal well-conserved sequences and, given its massive sequence variability, likely accommodates considerable tertiary and quaternary structural diversity despite maintaining consistent overall structural organization of each subunit (Allred et al., 2000; Al-Khedery and Allred, 2006; Brayton et al., 2007; Jackson et al., 2014). SGC occurs in both *ves1*α and *ves1*β genes (Al-Khedery and Allred, 2006), and VESA1 holoproteins are comprised of comparably variant VESA1a and 1b subunits. The variety that can be expressed, and thus the unique molecular space that can be sampled by the protein’s surface, is extremely large. Consistent with *in vitro* observations (O’Connor et al., 1999) one would anticipate that many- perhaps most- VESA1 variants are non-functional in cytoadhesion. However, the extreme diversity arising from the assembly of mosaic *ves* genes and proteins, and the correspondingly large molecular space that can be sampled results in selection of VESA1 isoforms capable of binding to one or more endothelial receptors. With the enhanced *in vivo* survival of cytoadhesive parasites and immunologic elimination of non-cytoadhesive parasites, episodic establishment of dominant variant populations differing in their adhesive specificities would be favored. Such extreme variability, coupled with repeated rounds of positive selection and amplification, is essentially the same mechanism as that underlying *in vivo* peptide phage display (Pasqualini and Ruoslahti, 1996; George et al., 2003), albeit on a larger structural scale, and is readily mimicked in the laboratory (O’Connor et al., 1999).

## Discussion

The survival of *B. bovis* relies upon DNA repair mechanisms resulting in creation of variant protein structures. Thus, there is a real need to understand these mechanisms and their ramifications. How could sequence patches recombine into transcribed *ves* genes to replace existing sequences by a non-HR process without affecting the donor? One possibility is that this feat might be accomplished by repair enzymes with the capacity to switch templates repeatedly during replication or repair, similar to the formation of immunoglobulin genes in birds (Nakazato et al., 2018). In the *B. bovis* genome a single Polζ DNA polymerase can be identified, orthologs of which have template-switching capacity in yeast and mammalian cells (reviewed in (Brayton et al., 2007; Northam et al., 2013; Martin and Wood, 2019)). Interestingly, among *Plasmodium* spp. the presence of a Polζ ortholog influences the extent of antigenic variation (Kirkman and Deitsch, 2020; Siao et al., 2020). Template-switching involving a second *ves* locus as template would be facilitated by close proximity among *ves* genes. Evidence consistent with such proximity was found in a limited chromatin-conformation capture assay (Wang et al., 2012). Similarly, organization of *P. falciparum var* genes into “bouquets” at the nuclear periphery was observed (Freitas-Junior et al., 2000), likely facilitating the cascade of crossover reactions occurring during repair of double-strand DNA breaks in the *var* gene family encoding PfEMP1 (Zhang et al., 2019).

**Figure 1:**
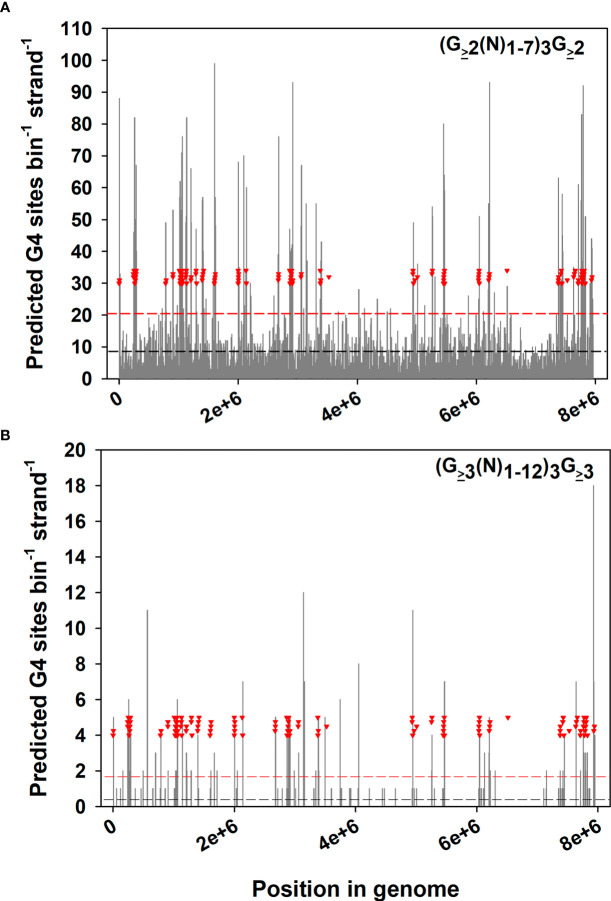
Distribution of predicted G-quadruplex sequences relative to *ves* genes in the *B. bovis* genome. The *B. bovis* C9.1 genome (Jackson et al., 2014) was concatenated and surveyed for G4 motifs, using **(A)** the low stringency motif (G_≥2_(N)1-7)_3_G_≥2_ or **(B)** a high-stringency motif (G_≥3_(N)_1-12_)_3_G_≥3_. These results were plotted as histograms of G4 density per 8 Kbp genome segments. Inverted red triangles indicate the “left”-most ends of *ves* coding sequences. The dashed horizontal lines represent the mean G4 density across the genome (black), or +2 s.d. (red).

How are SGC-mediated sequence changes focused on *ves* genes? As mentioned, *B. bovis* lives within an environment that experiences periodic high levels of oxidative stress. The guanine bases of G-G dinucleotide pairs present in G-quadruplex (G4) DNA are highly susceptible to oxidation, being readily damaged to give 8-OG (Fleming et al., 2017; Merta et al., 2019). A simple search of the *B. bovis* genome for sequences predicted to be competent to form G4 structure reveals that such sequences are highly enriched within or near (≤ 8 Kbp) *ves* loci (**Figure 1**). The full extent of enrichment is difficult to predict, as G4 structure may form with only two stacked G-quartets (**Figure 1A**), or may involve three or more (**Figure 1B**). Due to a need for DNA strand separation in order to form, actively transcribed DNA is far more prone to G4 formation than non-transcribed DNA (Rodriguez et al., 2012). This would have the effect of focusing oxidative damage and mutation on the *ves* family and specifically the LAT, and could act as a trigger for the SGC process. The G4 “focusing” effect is so strong that it serves to regulate promoter function in some DNA repair protein genes (Clark et al., 2012; Fleming et al., 2019). In concert with a second translesion polymerase, Rev1, DNA Polζ activity is important to lesion bypass of damaged bases such as 8-OG and stalled replication forks (Freisinger et al., 2004; Northam et al., 2013). Orthologs of both Rev1 and Polζ translesion polymerases are transcribed by asexual stage *B. bovis* parasites (Brayton et al., 2007; Pedroni et al., 2013). The significance of these components to SGC, antigenic variation, and cytoadhesion is not known, but we are pursuing this potential connection.

To summarize, hemoparasites live in a highly oxidative environment, and I suggest that cytoadhesion evolved to mitigate this damage. The *B. bovis* genome is greatly enriched in sequences with the potential to form G4 structure within and/or near *ves* genes. As G4 DNA is formed primarily within actively-transcribed sequences, can disrupt replication, and is overtly susceptible to oxidative damage this could make the LAT a sensitive target for damage. Repair of damage *via* a translesion polymerase with template-switching capabilities could both help to maintain overall genome integrity and result in local inclusion of ectopic sequence patches, the basis of SGC. Mosaic VESA1 protein isoforms created by SGC enable selection for adhesion, simultaneously avoiding immune recognition and oxidative damage. The ability to disrupt important mediators of SGC could not only affect parasite viability directly, but also diminish antigenic variation and cytoadhesion, reducing parasite survival and pathology.

## Data Availability Statement

Publicly available datasets were analyzed in this study. This data can be found here: https://www.sanger.ac.uk/resources/downloads/protozoa/babesia-bovis.html.

## Author Contributions

The author confirms being the sole contributor of this work and has approved it for publication.

## Funding

The author was supported by a University of Florida College of Veterinary Medicine intramural grant, account 00131124.

## Conflict of Interest

The author declares that the research was conducted in the absence of any commercial or financial relationships that could be construed as a potential conflict of interest.

## Publisher’s Note

All claims expressed in this article are solely those of the authors and do not necessarily represent those of their affiliated organizations, or those of the publisher, the editors and the reviewers. Any product that may be evaluated in this article, or claim that may be made by its manufacturer, is not guaranteed or endorsed by the publisher.
